# Frequency and duration of extreme hypoxemic and hyperoxemic episodes during manual and automatic oxygen control in preterm infants: a retrospective cohort analysis from randomized studies

**DOI:** 10.1186/s12887-022-03407-x

**Published:** 2022-06-17

**Authors:** Thomas E. Bachman, Wes Onland, Anton H. van Kaam, Karel Roubik, Helmut D. Hummler, Mithilesh Lal, Gianluca Lista, Carlos A. Fajardo

**Affiliations:** 1 Czech Technical University in Prague, School of Biomedical Engineering, Prague, Czech Republic; 2 Emma Children’s Hospital, Department of Neonatology, Amsterdam UMC, Amsterdam, Netherlands; 3 Amsterdam Reproduction and Development, Amsterdam, The Netherlands; 4 Department of Neonatology, University Hospital, Tuebingen, Germany; 5 James Cook University Hospital, Neonatology, South Tees NHS Trust, Middlesbrough, UK; 6 Vittore Buzzi Children’s Hospital, Neonatology, Milan, Italy; 7 Alberta Children’s Hospital, Neonatology, Calgary, Canada

**Keywords:** Oxygen saturation, Neonatology, Hypoxemic episodes, Hyperoxemia episodes

## Abstract

**Objective:**

Neonatal exposure to episodic hypoxemia and hyperoxemia is highly relevant to outcomes. Our goal was to investigate the differences in the frequency and duration of extreme low and high SpO_2_ episodes between automated and manual inspired oxygen control.

**Design:**

Post-hoc analysis of a cohort from prospective randomized cross-over studies.

**Setting:**

Seven tertiary care neonatal intensive care units.

**Patients:**

Fifty-eight very preterm neonates (32 or less weeks PMA) receiving respiratory support and supplemental oxygen participating in an automated versus manual oxygen control cross-over trial.

**Main measures:**

Extreme hypoxemia was defined as a SpO_2_ < 80%, extreme hyperoxemia as a SpO_2_ > 98%. Episode duration was categorized as < 5 seconds, between 5 to < 30 seconds, 30 to < 60 seconds, 60 to < 120 seconds, and 120 seconds or longer.

**Results:**

The infants were of a median postmenstrual age of 29 (28-31) weeks, receiving a median FiO_2_ of 0.28 (0.25-0.32) with mostly receiving non-invasive respiratory support (83%). While most of the episodes were less than 30 seconds, longer episodes had a marked effect on total time exposure to extremes. The time differences in each of the three longest durations episodes (30, 60, and 120 seconds) were significantly less during automated than during manual control (*p* < 0.001). Nearly two-third of the reduction of total time spent at the extremes between automated and manual control (3.8 to 2.1% for < 80% SpO_2_ and 3.0 to 1.6% for > 98% SpO_2_) was seen in the episodes of at least 60 seconds.

**Conclusions:**

This study shows that the majority of episodes preterm infants spent in SpO_2_ extremes are of short duration regardless of manual or automated control. However, the infrequent longer episodes not only contribute the most to the total exposure, but also their reduction in frequency to the improvement associated with automated control.

## Background

Continuous of monitoring of peripheral oxygen saturation (SpO_2_) is the standard of care for preterm infants requiring supplemental oxygenation. Keeping the SpO_2_ within an acceptable range and minimizing exposure to extremes is associated with better neonatal and long-term outcomes.

Titrating the fraction of inspired oxygen (FiO_2_) to maintain the SpO_2_ within a prescribed target range is a challenging task in daily care, resulting a mere 50% compliance within the intended target range [1]. As a result of disordered breathing and apnea, these infants frequently desaturate and caregivers sometimes respond with a transient increase in FiO_2_ to offset the potential hypoxemia. However, the response with an increase in FiO_2_ is often slow and inappropriate resulting in extended episodes of hypoxemia as well as swings to hyperoxemia. In addition, a failure to quickly reduce the FiO_2_ back to baseline levels once the desaturation has resolved further increases the risk of hyperoxemia [2]. The cumulative time in both extreme saturations is impacted by both the frequency and duration of these episodes. Previous studies have shown that the duration in extreme saturations is probably the key determinant for the risk of adverse outcomes [3, 4].

Given the limitations of manual FiO_2_ titration, automated FiO_2_ control algorithms for SpO_2_ have become available and studied. These studies have shown that automated control improves the time within the intended target range, and reduces time spend at the SpO_2_ extremes [5, 6]. It is unclear how automated FiO_2_ control impacts the frequency and duration of episodes of extreme hypoxemia and hyperoxemia. Some studies have reported a reduced frequency of one duration (1 min) associated with automated control [5]. More information is needed.

The aim of this post-hoc analysis was to provide additional detailed characterization of the frequency and duration of severe hypoxemic and hyperoxemic episodes, and specifically how they differ between automated and manual FiO_2_ control.

## Methods

This was a prospectively defined analysis of retrospective data from randomized cross-over studies investigating automated versus manual FiO_2_ control in preterm infants 32 weeks gestational age or less in need of invasive or non-invasive respiratory support. The study was carried out in accordance with relevant guidelines and requirements.

### Design

This is a post-hoc analysis of a cohort from prospective multicenter randomized cross-over studies [7–10].

### Setting

The 7 study sites were tertiary care neonatal intensive care units in 6 different countries.

### Subjects

Eligible subjects who were born less than 33 weeks gestational age were considered from four cross-over studies investigating manual versus automated FiO_2_ control (AVEA-CLiO2, Vyaire Mettawa IL, USA) in which individual subject data was available [7–10]. These studies used either two 24 or 12 hour intervention periods. To avoid potential bias, the two selection criteria were made prospectively, that is, without reviewing the outcomes. Since these individual studies used different SpO_2_ targets, and we did not want to confound the results, we selected only studies using a SpO_2_ mid-point of 90% and a target range width of 4%. This excluded two studies [9, 10]. Finally, subjects in the two remaining studies [7, 8] that did not spend at least 75% of time on supplemental oxygen were excluded to permit better characterization of hyperoxemic episodes. All these cases are from clinical trials with ethics approval, and are de-identified. All subjects were enrolled with written informed consent of their guardian(s).

### Outcome measures

We defined the primary SpO_2_ exposure metrics for SpO_2_ extremes as < 80% (hypoxemia) and > 98% (hyperoxemia). Episodes of 600 seconds or longer were excluded as being likely related to procedures and not representative of routine SpO_2_ management. The primary endpoint was the percent time of SpO_2_ episodes in each of 5 episode-duration categories (< 5 s, 5 to < 30s, 30 to < 60s, 60 to < 120 s, 120 to < 600 s). We also determined the frequency of episodes within each duration epoch. This categorization was based on data collected with a resolution of every 5 seconds, and two consecutive data points were defined as 5 seconds.

### Statistical analyses

With the sample of 58 subjects, we determined that we would have a > 80% chance of detecting an absolute difference of 10%-time, assuming an absolute variance of 25%-time, with an uncertainty of *p* < 0.05.

Extraction of the endpoints from the 5-second data points in the database was accomplished with purpose-built software (MatLab, Mathworks, Natick MA USA). Differences among the episode length categories and mode of FiO_2_ control were determined with the Kruskal-Wallis test with Dunn’s procedure for pairwise comparisons. A two-tailed p < 0.05 was considered statistically significant. Statistical tests were conducted with XLSTAT v19.03 software (Addinsoft, Paris, France).

## Results

From the initial 179 potential subjects, using the prospective selection criteria, we made the following exclusions: two studies (59 subjects) were excluded because of their target range [9, 10], 40 subjects were excluded from one study that evaluated high and low target range cohorts [7] and finally, due to higher exposure to room air, in the remaining two studies [7, 8], 22 subjects were excluded (12, 10 respectively). Thus, we evaluated the SpO_2_ control of 58 preterm infants receiving respiratory support and supplemental oxygen. Fifty-one percent of the cases came from one study [7], and the rest from the second study [8]. The inspired oxygen in these studies was controlled manually (M-FiO_2_) for 1 day and automatically (A-FiO_2_) on the other, in random order. All the infants were on the same mode of respiratory support (noninvasive or intubated) during the days of manual and automated control. Most were managed noninvasively (83%). The demographics of the subjects are shown in Table 1, they were mostly extremely preterm and were studied weeks after birth. The subjects’ baseline oxygen needs were relatively low (median FiO_2_ 0.28, IQR 0.25-0.32). A majority of the subjects spent no time on room air (59%). Those who did have periods without supplemental oxygen, spent a nominal amount of time without (median 4%, IQR 1-9%). The median SpO_2_ for both the automated and manual control methods were nearly identical (91%). However, there was a difference between the groups in the time spend outside normoxemia; the SpO_2_ was < 87% for 13% of the time during A-FiO_2,_ and 16% during M-FiO_2_, whereas 11% of the time the SpO_2_ was > 95% during A-FiO_2_, and 16% during M-FiO_2_. These differences were statistically significant (*p* < 0.001).

**Table 1:** Subject demographics

Birth Weight (grams)	805 (726–949)
Gestational Age at birth (weeks ^days^)	25^4^ (25^0^–26^3^)
Gestational Age at entry (weeks ^days^)	29^2^ (28^2^–31^0^)
Postnatal Age (days)	20 (15–29)
Gender (% male)	52

Presented as median and interquartile range (IQR), or fractional percent

There were frequent episodes at SpO_2_ extremes with differences between the two methods of control as shown in the Table 2. Episodes at the SpO_2_ extremes (< 80 and > 98%), tended to be less frequent during A-FiO_2_ within each of the 5 episode-duration categories. Most of these extreme episodes were shorter than 30 seconds.

**Table 2:** Frequency of extreme hypoxemic and hyperoxemic episodes

Episodes(/day)	Auto	Manual	*P*
< 80% SpO2			
Total	103(15–175)	216 (118–352)	< 0.001
< 5 s	42 (22–74)	67 (34–116)	< 0.05
5- < 30 s	45 (22–92)	61 (36–105)	ns
30- < 60 s	9 (3–17)	14 (9–23)	< 0.01
60 - < 120 s	1 (0–3)	6 (3–11)	< 0.001
120+ s	0 (0–1)	2 (1–4)	< 0.001
> 98% SpO2			
Total	24 (8–63)	75 (34–147)	< 0.001
< 5 s	11 (3–24)	23 (11–47)	ns
5- < 30 s	11 (4–29)	26 (12–63)	< 0.05
30- < 60 s	3 (0–8)	8 (3–17)	< 0.001
60 - < 120 s	0 (0–3)	3 (1–8)	< 0.001
120+ s	0 (0–1)	1 (0–4)	< 0.001

Hypoxemia is SpO_2_ < 80% and hyperoxemia SpO_2_ > 98%

Presented as median and interquartile range (IQR)

**Fig. 1:**
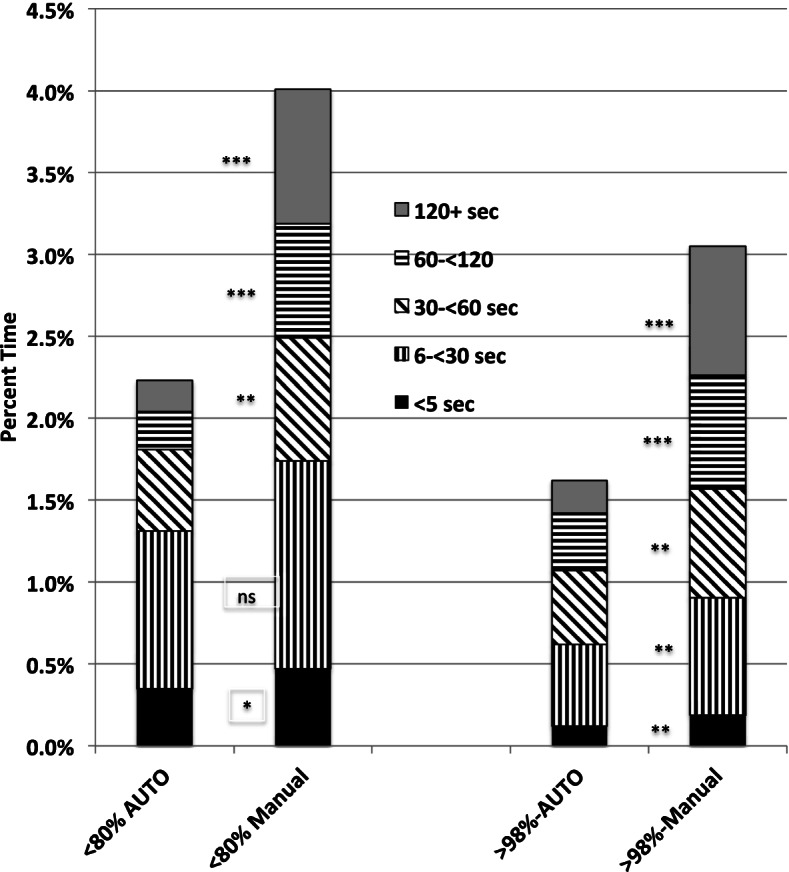
Stacked Histogram of % Time at SpO_2_ Extremes. Differences in % Time between automated fraction of inspired oxygen (A-FiO_2_) control and manual (M-FiO_2_) control: Total %Time (A-FiO_2_-M-FiO_2_):> 98% *p* < 0.001, < 80% < 0.0O1, For each of the 5 duration categories (ns, * < 0.05, ** < 0.01, *** < 0.001)

Our primary endpoint is shown in the Fig. 1. It presents the total time at< 80% and at > 98% SpO_2_, along with the contribution towards the total from each of the 5 episode-duration categories. The difference in the total time for A-FiO_2_ and M-FiO_2_ at each of these two endpoints was significantly different (*p* < 0.001), favoring A-FiO_2_. The time at each of the episode length categories tended to be lower during A-FiO_2_, and nearly all differences were statistically significantly significant [all but 5 to < 30 seconds with SpO2 < 80%]. Both shorter and longer episodes made a relevant contribution to the total duration of extreme SpO_2_ exposure with about half of the duration of time in hypoxemia < 80% resulted from episodes with a duration of 30 seconds or shorter, whereas one third of the duration of time with hyperoxemia > 98% was a result of episodes 30 seconds or shorter. In contrast, the duration of time < 80% from episodes 60 seconds or longer were 19% for A-FiO_2_ and 49% for M-FiO_2_. More than a third of the duration of time > 98% were from episodes 60 seconds or longer (34% A-FiO_2_, 38% M-FiO_2_). Importantly, though infrequent during both modes of control, nearly two-thirds of the reduction of total time spent at the extremes between automated and manual control (3.8 to 2.1% for < 80% SpO_2_ and 3.0 to 1.6% for > 98% SpO_2_) was from a reduction of episodes of 1 min or longer.

## Discussion

In a population cohort of extremely preterm infants receiving supplemental oxygen and respiratory support, we investigated the frequency and duration of marked hypoxemic and hyperoxemic episodes during both A-FiO2 and M-FiO2. We confirmed that the infrequent longer episodes were the primary contributor to total time at SpO_2_ extremes. We found that, compared to M-FiO2, automated control of SpO_2_ reduced the number of episodes in all durations, but that most of the reduction in the total exposure to these extremes was from a reduction of episodes of 1 min or longer.

Though we provide more detail, our hypoxemic episode results are consistent with other studies. The extensive report of Poet et al., found that hypoxemic exposure with manual FiO_2_ control is comprised of episodes of desaturations across a range of durations, with prolonged episodes being infrequent, but nevertheless contributing markedly to the total exposure to SpO_2_ less than 80% [3]. Other studies using this automated FiO_2_ control algorithm have reported a reduced frequency of hypoxemic episodes longer than a minute, compared to manual FiO_2_ control [9, 11]. Our study identified that the duration of hypoxemic episodes is the primary factor in A-FiO_2_ reducing total time in hypoxemia. One study of A-FiO2 reported that the total number of shorter episodes below the target range was increased but that the longer episodes were decreased with A-FiO_2_, suggesting that potentially longer episodes were compressed [9]. This was not confirmed in our study. Rather we found that automated FiO_2_ control also reduced the frequency of shorter episodes of hypoxemia. We suggest that this different finding is a result of a higher median SpO_2_ during manual in the previous report. The impact of such a shift is consistent with another study [7].

We believe our data are the first report of the details of the frequency and duration of episodes of hyperoxemia. We found, consistent with desaturations, that it is comprised of episodes across a range of duration, with prolonged episodes being infrequent, but nevertheless contributing markedly to the total exposure to SpO_2_ greater than 98%. Studies of this automated FiO_2_ control system have also shown a reduction of the frequency of hyperoxemic episodes longer than a minute [8–11], but our study demonstrated that this is the primary factor in reducing total time in hyperoxemia with A-FiO_2_.

There is limited information on how these differences might impact outcome, that has come from rigorous post-hoc analyses of the large randomized trials. One report found that better control of the SpO_2_, regardless of the target range, improved long term outcomes but they did not report the associated exposure to SpO_2_ extremes [12]. Another reported an association between cumulative exposure to SpO_2_ < 80%, which correlated to the number of prolonged episodes, with an increased risk of late death or disability at 18 months and also speculated that therapies that reduced these prolonged events could improve long-term outcomes [3]. Another research team reported that an increased frequency of all hypoxemic events was associated with severe retinopathy of prematurity and bronchopulmonary dysplasia [13, 14]. We speculate that more frequent desaturations would also correlate with more prolonged hypoxemic episodes, as well as an increased risk of overshoot to hyperoxemia. The impact of hyperoxemia has not been so carefully evaluated, but a landmark study published nearly 20 years ago confirmed that high levels of SpO_2_ are associated with severe retinopathy of prematurity and bronchopulmonary dysplasia, without a difference in hypoxemia [4]. Regardless, it is not clear whether these studies report a cause-effect relationship, or rather a marker of adverse outcome.

Alarm setting strategies ought to be studied much more. They should strive to reduce the time at SpO_2_ extremes, by specifically balancing the risk of missing important events because of false negatives versus not responding to important events due to alarm fatigue. These results may help in formulating strategies to reduce oximeter alarm fatigue by considering the trade-off between false-positive and false-negative alarms that is associated with the alarm delay. Our data show that during manual FiO_2_ control, most of the episodes of extreme SpO_2_ levels in this group of infants resolved on their own in less than 30 seconds. One might question the clinical relevance of the settings that trigger highly frequent alarms not needing attention. Nevertheless, these frequent alarms might be useful in alerting nurses to instability. In contrast, it seems reasonable that an A-FiO_2_ control system, which continuously makes FiO_2_ adjustments as often as many times per minute, might need different alarm delays. It is important to note that in the case of A-FiO_2_ control, a saturation alarm indicates that changes in the FiO_2_ have not adequately mitigated the alarm condition, and thus personal attention is needed. Our data suggest that alarms delay during A-FiO2 might be set at 60 seconds or longer in order to reduce false alarms. One study supports these considerations [15].

We evaluated one A-FiO2 system and the findings of this study should be generalized to other A-FiO2 systems cautiously. Alternative algorithms, that consider changes much less frequently than every second would not be expected to reduce episodes of shorter duration. Nevertheless, we also found that much of the reduction of total exposure was a result of reducing the duration of longer episodes, so this might not be clinically relevant. In contrast more frequent automatic adjustments might lead to more overshoot between extremes, which is a constant concern during manual control. Overshoot using the A-FiO2 algorithm that we studied has shown to be better than M-FiO_2_ [9, 16], but this needs to be evaluated in other A-FiO2 systems, regardless of their frequency of adjustment.

The primary limitation of our study is that it reflects a small population of infants and a single day of exposure. However, the results do seem to be consistent with other studies. The thresholds for extreme levels of SpO_2_ were prospectively defined, and have been shown to be associated with extreme levels of PaO_2_ [17]. Nevertheless, evaluation of other_,_ less extreme, SpO_2_ thresholds might yield different conclusions and also impact outcomes. Nevertheless in a post hoc analysis we found the effects of using cut offs of < 87% and > 95% were similar, suggesting the findings would be insensitive to the exact cut off. Further we counted episodes > 98% even if the subject was temporarily not receiving supplemental oxygen. This would tend to increase the frequency of such episodes, but our analysis population, had a very limited amount of time without supplemental oxygen. There are also some other aspects of the study impacting the generalization of our work. First, our study reflects experience with a lower SpO_2_ target range resulting in a median SpO_2_ of 91%. A higher target range, if resulting in a higher median SpO_2_, would likely have a different profile. One study comparing two ranges (89-93% and 91-95% SpO_2_) found a shift to less hypoxemia and more hyperoxemia during both automated and manual FiO_2_control [8]. Studies also suggest that M-FiO_2_ control of SpO_2_ at higher target ranges is easier to manage than at lower target ranges [7, 18]. Finally, this study population was not large enough to explore relative differences associated with invasive and noninvasive ventilation or during periods of differing stability.

## Conclusions

Our study characterizes the distribution of extreme hypoxemic and hyperoxemic episodes. Nearly all of these extreme episodes are of short duration. However, the relatively infrequent longer episodes were the main contributors to the total SpO_2_ extreme exposure. We demonstrated that A-FiO2 results in fewer episodes of all durations, but that a reduction in the infrequent longer episodes was the main factor in its improved effectiveness. We believe this information might be useful not only in refining SpO_2_ alarm practices, but also for refining and comparing the effectiveness of A-FiO2 control systems.

## Data Availability

The data sets analyzed in this study are not publicly available, as a result of the investigator agreements when conducted, but are available from the corresponding author upon reasonable request.

## Abbreviations

SpO_2_: Peripheral oxygen saturation
FiO_2_: Fraction of inspired oxygen
IQR: Interquartile range
A-FiO2: Automated control of FiO_2_
M-FiO2: Manual control of FiO_2_
